# An Autographa californica multiple nucleopolyhedrovirus-encoded microRNA, AcMNPV-miR-5, downregulates the expression of viral gene ac66

**DOI:** 10.1099/jgv.0.002155

**Published:** 2025-09-30

**Authors:** Tingkai Teng, Zhuowen Duan, Ang Li, Jinwen Wang

**Affiliations:** 1 School of Life Sciences, Sun Yat-sen University, Guangzhou 510275, PR China

**Keywords:** baculovirus, microRNA (miRNA), regulation, target gene

## Abstract

Four Autographa californica multiple nucleopolyhedrovirus (AcMNPV)-encoded microRNAs (miRNAs) have been characterized previously. Here, we report the fifth AcMNPV-encoded miRNA, AcMNPV-miR-5 (Ac-miR-5), which downregulates the viral gene *ac66*. Target genes were predicted through sequence analysis and validated using luciferase reporter assays. The regulatory effects of Ac-miR-5 on *ac66* expression were assessed by reverse transcription quantitative PCR and Western blot. The impact of Ac-miR-5 overexpression on virus infection was analysed by TCID_50_ assay and quantitative real-time PCR in Sf9 cells. The results showed that Ac-miR-5 downregulates *ac66* at both the mRNA and protein levels. Meanwhile, the budded virion production and DNA replication were decreased. Furthermore, microscopy revealed a decrease in the number of polyhedra formed. These findings suggest that Ac-miR-5 overexpression restricts viral load, potentially contributing to the establishment of a stable viral infection within the host cell.

**Impact statement**

Autographa californica multiple nucleopolyhedrovirus (AcMNPV) encodes several miRNAs that modulate viral infection. Building on our previous work characterizing four such miRNAs (Ac-miR-1 to -miR-4), this study characterizes Ac-miR-5, which targets the viral gene *ac66*. We demonstrate that Ac-miR-5 downregulates *ac66* expression. Ac-miR-5 overexpression can lead to reduced budded virus production, DNA replication and polyhedra formation. These results suggest that Ac-miR-5, like other AcMNPV miRNAs, contributes to viral load control, potentially facilitating persistent infection. This research provides further insight into the complex interactions between baculoviruses and their hosts and may open new avenues for antiviral development.

## Data Summary

The authors confirm that all supporting data and protocols have been provided within the article or through supplementary data files.

## Introduction

MicroRNAs (miRNAs) are small, non-coding RNAs that play crucial roles in post-transcriptional gene regulation. By binding to target mRNAs, they induce either mRNA degradation or translational repression, thereby influencing a wide range of cellular processes, from development and differentiation to immune responses and disease pathogenesis [1,2]. While many miRNAs are encoded by the host genome, viruses have also evolved to utilize this powerful regulatory mechanism. Virus-encoded miRNAs, transcribed from viral genomes, can target both viral and host mRNAs, modulating viral replication and manipulating host cell functions to facilitate infection [3,5].

Baculoviruses are a family of large, double-stranded DNA viruses that infect insects and produce two kinds of virion, budded virus (BV) and occlusion-derived virus (ODV), during their infection cycle [6]. They are widely used as biological control agents in agriculture and also serve as a valuable model system for studying virus–host interactions. Phylogenetically, baculoviruses are categorized into four genera: *Alphabaculovirus*, *Betabaculovirus*, *Gammabaculovirus* and *Deltabaculovirus*. Alphabaculovirus is further subdivided into Group I and Group II [7]. AcMNPV (Autographa californica multiple nucleopolyhedrovirus), a Group I alphabaculovirus, is a well-established model for understanding baculovirus molecular biology and pathogenesis [6,7]. Several baculoviruses, including AcMNPV, Bombyx mori nucleopolyhedrovirus (BmNPV) and Spodoptera litura nucleopolyhedrovirus, encode their own repertoire of miRNAs [8,10], which have diverse functions impacting both the viral life cycle and the host cellular environment [11,17]. Four AcMNPV-encoded miRNAs have been previously characterized [12,14,17]. Overexpression of these miRNAs can reduce infectious BV production. Specifically, Ac-miR-1 decreases viral DNA replication and accelerates polyhedra formation by primarily downregulating viral genes *ac94* and *ac95* and upregulating *ac18* [12,14]; Ac-miR-2 also diminishes viral DNA replication but delays polyhedra formation, mainly targeting viral genes *ac28*, *ac37*, *ac49* and *ac63* [17]; Ac-miR-3 accelerates ODV formation by primarily downregulating *ac101* and several other viral genes [15]; and Ac-miR-4 delays ODV embedding into polyhedra and extends cell lifespan by primarily targeting the host gene *alg-2* [16].

In this study, we characterize AcMNPV-miR-5, the fifth identified AcMNPV-encoded miRNA. Located in the 5′ region of *ac-49k* and *ie-01*, Ac-miR-5 is expressed at 6 and 12 h post-infection (h p.i.), reaching maximum expression at 24 h p.i. [8]. We demonstrate that the viral gene *ac66* is a target of Ac-miR-5 and is downregulated by this miRNA. Overexpression of Ac-miR-5 leads to decreased BV production, reduced viral DNA replication and a decrease in the number of polyhedra formed.

## Methods

### Viruses and cell lines

The bacmid bMON14272 (Invitrogen), which contains an AcMNPV genome, was maintained in DH10B cells [18]. *Spodoptera frugiperda* IPLB-Sf21-AE clonal isolate 9 (Sf9) insect cells were maintained in Grace’s medium (Invitrogen Life Technologies) containing 10% FBS at 27 °C. 293T cells were maintained in Dulbecco’s modified Eagle’s medium (Hyclone) containing 10% FBS at 37 °C with 5% carbon dioxide. The viral inoculum was allowed to adsorb to cells for 1 h of infection, or the bacmid was applied to cells for 4 h of transfection at 27 °C. Time zero was defined as the time when the viral inoculum was replaced as described previously [19].

### Cell transfection

Approximately 10^6^ cells were plated in a six-well plate overnight prior to transfection; 1 pmol of mimic/inhibitor (RiboBio) and vector was transfected using Cellfectin (Gibco) in Sf9 cells or Lipofectamine 2000 (Invitrogen) in 293T cells. Total RNAs were isolated using the TRIzol method (Invitrogen).

### Stem-loop real-time PCR for Ac-miR-5 analysis

RNA samples were extracted using TRIzol reagent (Invitrogen). miRNAs were reverse-transcribed using a stem-loop primer and the miRNA First Strand cDNA Synthesis Kit (Vazyme) in a 20 µl reaction mixture. The remaining RNAs were reverse-transcribed using ReverTra Ace qPCR RT Master Mix with gDNA Remover (TOYOBO). The reverse transcription product was used to perform quantitative real-time PCR (qPCR) with a real-time PCR detection system (QuantStudio 6 Flex; Thermo Fisher). A 10 µl qPCR mixture contained 1 µl of cDNA, 5 µl of 2× SYBR qPCR Master Mix, 0.2 µl of forward or reverse primers (10 µM) and nuclease-free water to make up the total volume. PCR was conducted on the QuantStudio 6 Flex system with a 30 s denaturation at 95 °C, followed by 40 cycles of 10 s at 95 °C and 30 s at 60 °C. Then, a program of 95 °C for 15 s, 60 °C for 60 s and 95 °C for 15 s was used to obtain melting curves. The reverse transcription quantitative PCR (RT-qPCR) data for each sample were calculated using the 2^-ΔΔCT^ (where CT is threshold cycle) method [20]. A 5S rRNA reverse transcript that was generated from random primers was used as a reference. Each reaction was performed in at least three independent experiments in triplicate.

### Prediction of target genes

The genome sequence (FASTA format) and genome annotation files (gff format) of AcMNPV were downloaded from NCBI. The transcriptome file of AcMNPV was extracted from the genome sequence file using the gffread software. Target genes of Ac-miR-5 were predicted using the miRanda program. The parameters are set as follows: the lowest score is 140, and the highest thermodynamic energy is −1.0 kcal mol^−1^, allowing G-U matching [21].

### Dual-luciferase reporter assay

The predicted candidate targets were evaluated to determine whether Ac-miR-5 regulates them by dual-luciferase assay. The predicted binding site region of each gene for Ac-miR-5 was cloned downstream of the *Renilla* luciferase gene in the psiCHECK-2 vector (Promega), as previously described [14,17]. Cel-miR-239b-5p, a miRNA from *Caenorhabditis elegans*, was used as negative control (NC) mimic (Table S1, available in the online Supplementary Material).

An unrelated (non-host) cell line, 293T cells, was used to verify the predicted targets to filter other irrelevant possible interactions between the virus and host. The cells were co-transfected with 0.1 µg of each reconstructed plasmid and 50 nM miRNA mimic using Lipofectamine 2000 (Invitrogen) according to the manufacturer’s instructions. The cells were then used to perform a dual-luciferase assay at 48 h post-transfection (h p.t.) using a dual-luciferase reporter assay system (Promega), as previously described [14,17]. The *Renilla* luciferase activity was normalized against the firefly luciferase activity, which was mediated by an independent promoter in the psiCHECK-2 vector.

### Construction of the Flag-tagged candidate target gene recombinant vectors

Take pdflag +*ac66* as an example. The sequences of the HSV TK promoter and SV40 poly(A) signal were obtained by PCR from the psiCHECK-2 plasmid, and the middle sequence of the Flag tag was obtained by annealing primers. Then, seamless cloning was used to connect these sequences into the pUC18 plasmid digested by EcoRI and BamHI to form the pUC18 +hlucFlag plasmid. The ‘CMV promoter +Flag tag +hGH tail signal’ sequence was obtained by PCR from the p3xFlag-CMV10 plasmid. Also, seamless cloning was used to connect the sequence into the pUC18 +hlucFlag plasmid digested by BamHI to form a pdflag plasmid. The protein-coding sequence of *ac66* was amplified from AcMNPV bacmid by PCR and subsequently combined into the pdflag plasmid, which was digested by NotI and BamHI to form the pdflag +*ac66* vector. The primers used were synthesized from RiboBio (Table S1).

### Construction of recombinant viruses overexpressing Ac-miR-5

The fragment containing Ac-miR-5 precursor sequence and specific restriction sites was amplified from AcMNPV bacmid by PCR and then ligated downstream of the IE-1 promoter and upstream of the SV40 poly(A) sequence in pBlueScript plasmid to generate the pBlue-1×-GP. To generate the pBlue-2×-GP, one copy of the Ac-miR-5 precursor fragment was digested with BamHI and PstI and ligated into the pBlue-1×-GP that had been digested with BglII and PstI. The pBlue-4×-GP and pBlue-8×-GP plasmids were constructed similarly. Then, the fragments containing IE-1 promoter, 1×/2×/4×/8× Ac-miR-5 precursor sequence and SV40 poly(A) sequence were digested by SacI and XhoI and ligated into the pFastBac1-PH-GFP (pFB1-GP) [where PH (P) represents polyhedrin and GFP (G) represents green fluorescent protein gene] to generate donor plasmid pFB1-1×/2×/4×/8×-GP. These donor plasmids were transformed into DH10B cells, respectively. As a result, five overexpression recombinant viruses – vAc^PH-GFP^ (WT), vAc^miR-5-1×-PH-GFP^ (1×), vAc ^miR-5-2×-PH-GFP^ (2×), vAc^miR-5-4×-PH-GFP^ (4×) and vAc^miR-5-8×-PH-GFP^ (8×) – were generated by the BAC-to-BAC technique system.

### Construction of the HA-tagged overexpressing Ac-miR-5 recombinant viruses

The ET recombination technique was used to add haemagglutinin (HA) on *ac66* in the above-constructed recombinant viruses. First, the linear US-HA-SV40-CmR-SV40-DS (U: upstream; S: homologous sequence; D: down) fragment was constructed as previously described [14,17]. Next, to facilitate homologous recombination, five types of DH10B cells (containing the WT, 1×, 2×, 4× and 8× bacmid) were co-transformed with pCasT plasmids. The resulting clones were induced by adding l-arabinose to achieve competence and then electro-transformed with 1 µg of the purified US-HA-SV40-CmR-SV40-DS fragment. Finally, the recombinant viruses with an HA tag on *ac66* were generated. The vA^ac66-HA-miR-5-4×-PH-GFP^ (4×-66:HA) and WT vA^ac66-HA-PH-GFP^ (WT-66:HA) are used in the following relevant overexpression experiments after stability and effectiveness tests.

### Time-course analysis of BV titres and production

Sf9 cells were infected with Ac-WT and Ac-miR-5-4× viruses at an MOI of 10 p.f.u./cell, respectively. Supernatants containing BVs were harvested at the designated time post-infection, and the BV titres were determined in triplicate by the TCID_50_ endpoint dilution assay [19]. Moreover, genomic DNA was extracted from 200 µl supernatants using an E.Z.N.A. Viral DNA Kit (OMEGA). Then, the DNA was extracted to perform qPCR amplification using a primer for *gp41*. The concentration of viral DNA genome copies within each sample was calculated using a standard curve generated from a dilution series of AcMNPV bacmid DNA.

### Quantitative analysis of viral DNA replication

To analyse viral DNA replication, qPCR was performed. Sf9 cells were infected with Ac-WT and Ac-miR-5-4× viruses at an MOI of 10 p.f.u./cell, respectively. Cells were harvested at the designated time post-infection. The total DNA in each sample was purified using a Genomic DNA Extraction Kit (OMEGA) and resuspended in 75 µl of double-distilled H_2_O. Then, 3 µl of DNA was taken to perform qPCR using SYBR Premix Ex Taq II (TaKaRa), and primers for *gp41*, a gene unique to AcMNPV, were used for amplification.

### RNA isolation and RT-qPCR

To analyse the mRNA expression levels of some related genes, Sf9 cells (2×10^6^) were infected with Ac-WT or Ac-miR-5-4× viruses at an MOI of 10 p.f.u./cell. Then, the cells were collected at designated time points, and RNAs were extracted with TRIzol reagent (Invitrogen). The RNAs were reverse-transcribed using a kit of RT Master Mix with gDNA remover (TOYOBO) in 10 µl reaction mixtures according to the manufacturer’s instructions. The reverse transcription product was used to perform qPCR using specific primers (Table S1) for specific genes, including *ac66*, *polyhedrin* (*polh*), *p10* and *spod-11-tox*. The qPCR was conducted as described above. The 2^-ΔΔCt^ method was used to calculate the expression level of each sample [19]. A 5S rRNA reverse transcript was usually used as a reference.

### Transmission electron microscopy

Sf9 cells (2×10^6^) were infected with Ac-WT or Ac-miR-5-4× viruses at an MOI of 10 p.f.u./cell. At 48 and 72 h p.i., cells were harvested and centrifuged at 1,000 ***g*** for 10 min. Then, the pelleted cells were fixed, dehydrated, embedded, sectioned and stained as described previously [22]. Samples were observed under a JEM-1400Flash (JEOL Ltd.) transmission electron microscope at an accelerating voltage of 120 kV.

### Western blot assay

To detect the protein expression levels of candidate targets, 293T cells were transfected with NC or Ac-miR-5 mimics, and Sf9 cells were infected with Ac-WT or Ac-miR-5-4× viruses (MOI of 10 p.f.u./cell). After a designated time, cells were washed twice with PBS and lysed with 25 µl radioimmunoprecipitation assay buffer (Yeasen). Then, lysates were spun at 10,000 ***g*** for 10 min after incubating on ice for 30 min. The supernatant proteins were collected and boiled at 95 °C for 10 min with 5× protein loading buffer (CWBIO). Equal volumes of each sample were resolved on a 15% SDS-PAGE and transferred to PVDF membranes with a semi-dry transfer cell (Trans-Blot SD; Bio-Rad), followed by blocking in 5% non-fat milk for 2 h at room temperature. The membrane was then probed with a monoclonal Flag (1 : 1,000; Beyotime) or HA antibody (1 : 2,000; Cell Signaling Technology) overnight at 4 °C according to the manufacturer’s instruction. Moreover, the reference proteins acting as controls were probed with monoclonal GAPDH antibody (1 : 3,000; Cell Signaling Technology). A goat anti-mouse antibody (1 : 1,000; Beyotime) conjugated to HRP was used as the secondary antibody. The blotting bands were visualized using an enhanced chemiluminescence (Beyotime) through Amersham Imager 600 (GE Healthcare Life Sciences) imaging system according to the manufacturer’s instructions.

### Statistical analyses

Statistical analyses were performed using GraphPad Prism software version 10.4.0 (GraphPad Software, Inc.). Statistical significance was calculated using Student’s two-tailed unpaired or multiple t tests, as well as two-way ANOVA. The Mann–Whitney U test in R was used for the statistical difference analysis of Table 1. *P<0.05; **P<0.01; ***P<0.001; ****P<0.0001.

**Table 1.** The percentage of cells containing polyhedra observed under light microscope

	% of cells containing polyhedra		
	24 h p.i.	48 h p.i.	72 h p.i.
Ac-WT	46±1	48±1	65±5.69
	*P*=0.033	*P*=0.033	*P*=0.1
Ac-miR-5-4×	22.33±0.577*	35±5.77*	53±2.52

The percentage of cells containing polyhedra was calculated by randomly choosing 100 infected cells for each field of view. For each sample, three fields of view were selected each time. The values were derived from the mean of the nine field counts. The counting was performed in triplicate for three independent experiments. Statistical significance was calculated using the Mann–Whitney U test, *P<0.05.

## Results and discussion

### Ac-miR-5 is conserved in alphabaculovirus

**Fig. 1.**
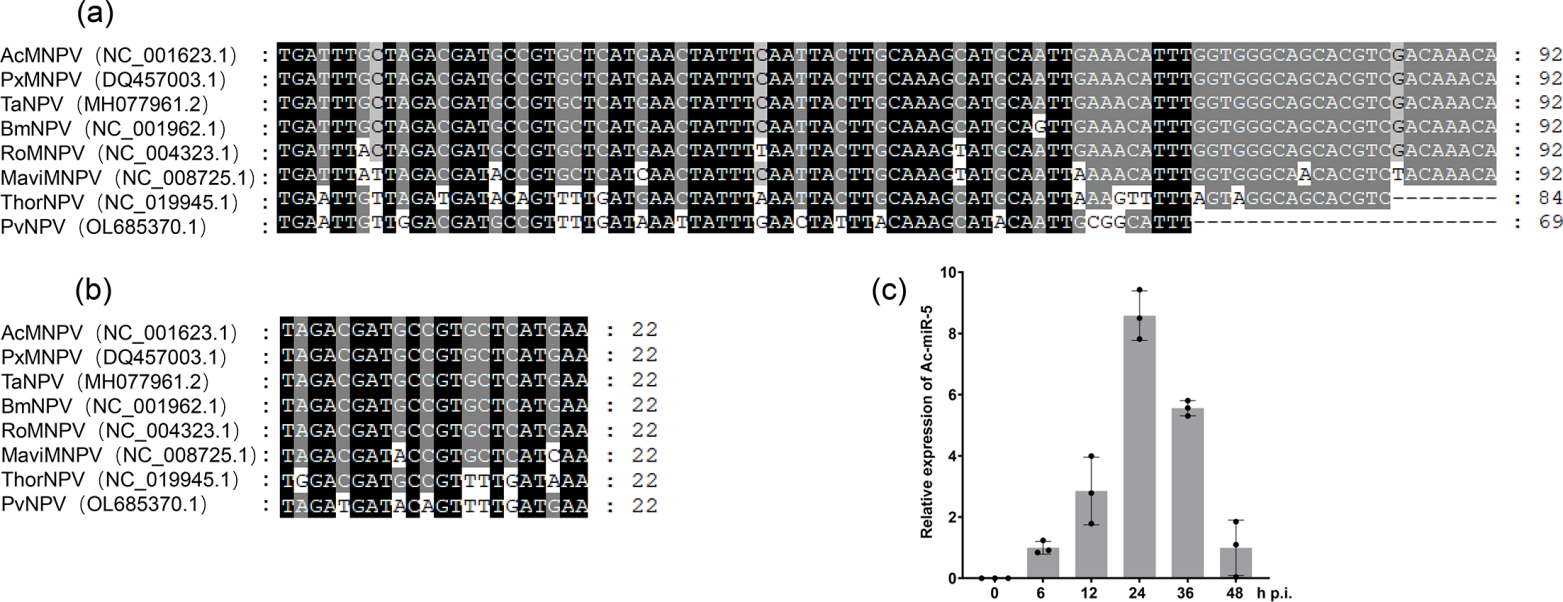
Conservative and expression phase analysis of Ac-miR-5. Conservative analysis of the precursor sequence (**a**) and mature sequence (**b**) of Ac-miR-5 in baculoviruses by NCBI Basic Local Alignment Search Tool and ClustalW. (**c**) The expression phase analysis of Ac-miR-5 by RT-qPCR. Columns represent the fold change of Ac-miR-5 expression. RNA samples were isolated from Sf9 cells at the indicated time points (10 p.f.u./cell). The expression level at each time point was normalized against that at 0 h p.i. 5S rRNA was used as an endogenous control. Each reaction was performed in triplicate in at least three independent experiments.

Ac-miR-5 is a 22 nt miRNA derived from a 92 nt precursor [8]. Sequence alignment revealed high conservation of both the mature and precursor sequences within the *Alphabaculovirus* genus, particularly in Plutella xylostella multiple nucleopolyhedrovirus (PxMNPV), Thaumetopoea pityocampa nucleopolyhedrovirus (TaNPV), BmNPV and Rosalia multiple nucleopolyhedrovirus (RoMNPV) (Fig. 1a, b). The mature sequence exhibited greater conservation, likely due to its shorter length (22 nt).

Our previous study demonstrated that Ac-miR-5 exhibits distinct expression at 6, 12, 36 and 48 h p.i., with peak expression at 24 h p.i. [8]. We confirmed this expression profile in the current study using stem-loop RT-qPCR, with results consistent with our previous findings (Fig. 1c).

### Viral gene *ac66* is a target of Ac-miR-5

**Fig. 2.**
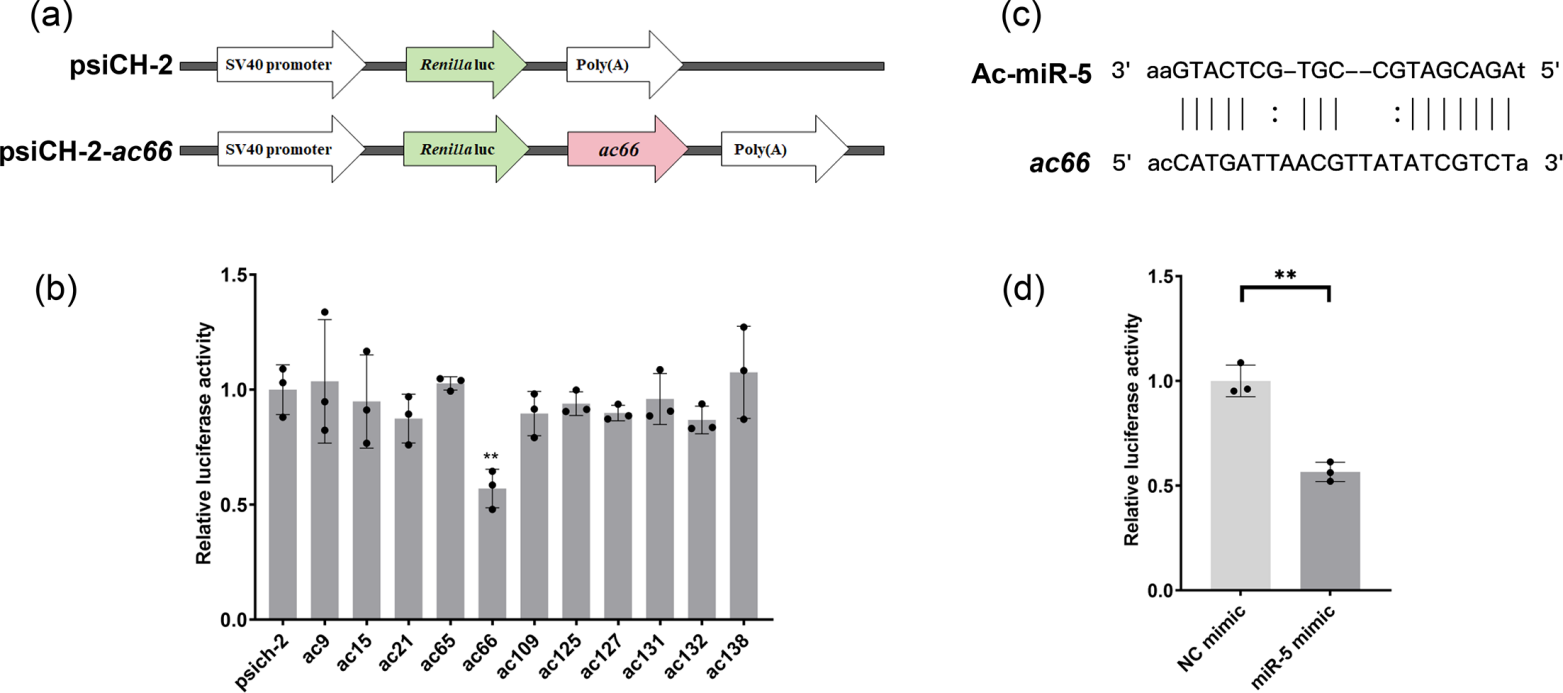
Dual-luciferase reporter assay tests the regulation of Ac-miR-5 on the predicted target sequences. (**a**) Schematic of luciferase reporter constructs for target gene testing, using *ac66* as an example. The reporter constructs contain the predicted target gene binding sequences inserted downstream of the *Renilla* luciferase (*luc*) gene. (**b**) The results of the dual-luciferase reporter assay of *ac66*, *ac9*, *ac15*, *ac21*, *ac65*, *ac109*, *ac125*, *ac127*, *ac131*, *ac132* and *ac138*. The activities of the reporter (psich-2) were detected as controls. (**c**) The binding site sequences of *ac66* for Ac-miR-5. (**d**) The luciferase reporter activity assay of *ac66*. The NC mimic was used as a negative control. The luciferase activities were detected at 48 h p.t. The data are presented as means and sd of three independent experiments. ns, no significant; *P<0.05; **P<0.01.

To identify potential targets of Ac-miR-5, we analysed the AcMNPV transcriptome and genome sequences using miRanda. Eleven viral genes were selected as candidate targets based on seed region pairing, minimum free energy (MFE) and miRanda score (Table 2). These candidates were then screened using a dual-luciferase reporter assay, employing *Renilla* luciferase as the reporter and firefly luciferase as the reference. This assay revealed that only *ac66* expression was distinctly downregulated by the Ac-miR-5 mimic; the other ten predicted genes showed no significant change (Fig. 2b). This specific targeting of *ac66* was further confirmed in a separate reporter assay, which demonstrated significant downregulation of luciferase activity by the Ac-miR-5 mimic compared to the NC mimic (Fig. 2d). These results indicated that viral gene *ac66* is a target and can be downregulated by Ac-miR-5.

**Table 2.** The predicted candidate target genes of Ac-miR-5 in AcMNPV

Accession no.	Candidate target	Score	MFE (kcal mol^−1^)	Binding site (nt)
NP_054038.1	*ac9*	155.00	−24.24	1108–1132
NP_054044.1	*ac15*	140.00	−16.92	898–919
NP_054050.1	*ac21*	148.00	−15.60	914–934
NP_054095.1	*ac65*	147.00	−18.56	1236–1257
NP_054096.1	*ac66*	154.00	−22.74	610–634
NP_054139.1	*ac109*	145.00	−19.91	908–931
NP_054155.1	*ac125*	140.00	−22.69	311–335
NP_054157.1	*ac127*	143.00	−20.98	342–363
NP_054161.1	*ac131*	159.00	−23.78	411–433
NP_054162.1	*ac132*	152.00	−14.55	409–430
NP_054168.1	*ac138*	154.00	−17.21	46–66

The score and MFE were calculated by miRanda, version 3.3a. The score represents the possibility of the target gene using the bioinformatics method. The binding site was counted from the 5′ end of the target gene sequence searched in the NCBI database.

**Fig. 3.**
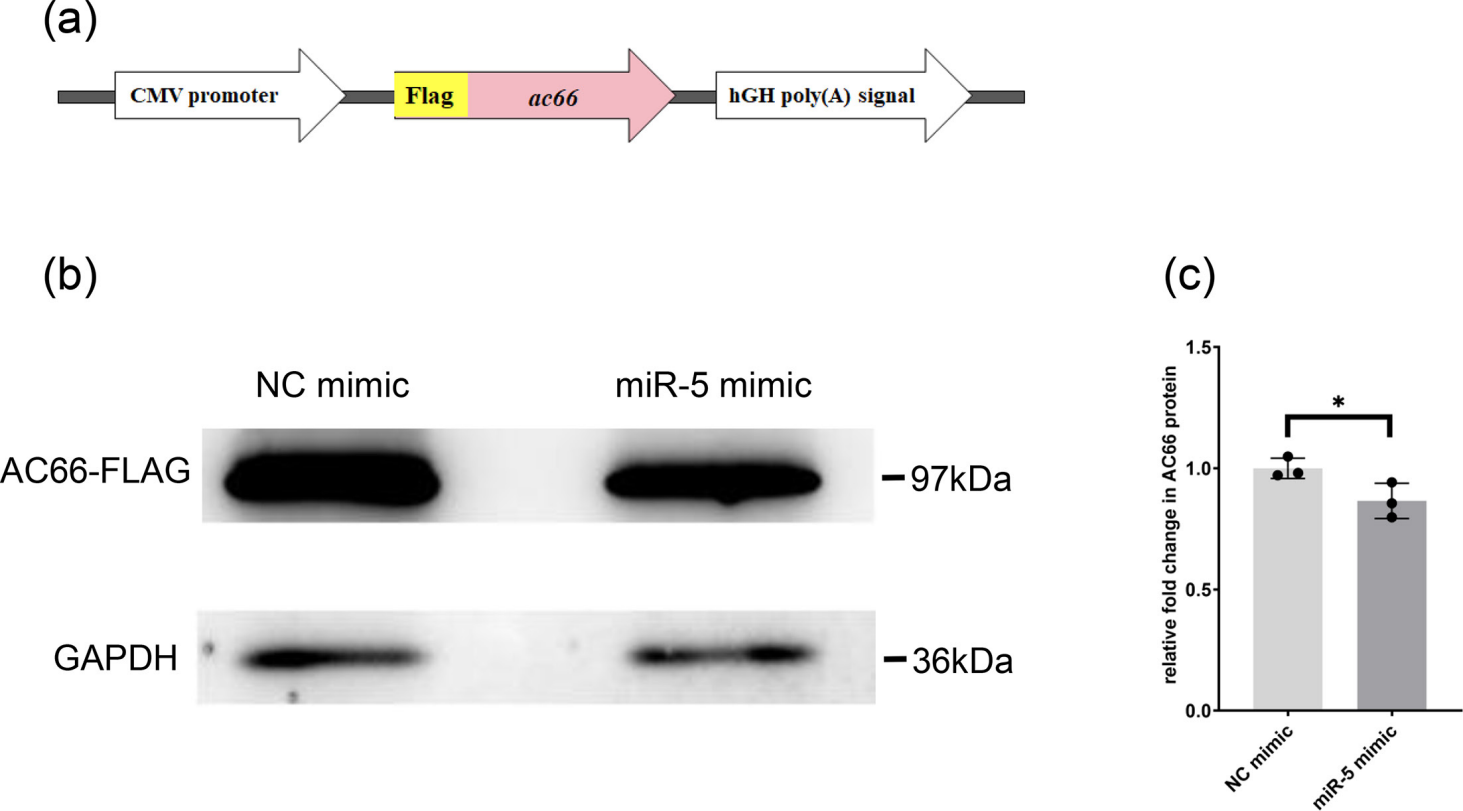
Western blot analysis to assess the regulatory effects of Ac-miR-5 on AC66 in 293T cells. (**a**) Illustrating the construction of pdflag+*ac66* recombinant expression plasmid. (**b**) Protein expression levels of *ac66* when 293T cells were administered with mimic miRNAs. (**c**) ImageJ quantification analysis of the Western blotting results. GAPDH represents a control protein. *P<0.05.

Furthermore, to assess the targeting effect of Ac-miR-5 on *ac66* protein expression, we performed Western blot analysis in 293T cells, a non-host cell line used to minimize potential interference. A Flag-tagged *ac66* expression vector was constructed by cloning the *ac66* CDS into the pdflag vector (Fig. 3a) [17]. 293T cells were co-transfected with the pdflag-*ac66* vector and either the Ac-miR-5 mimic or the NC mimic. Protein samples were collected 48 h p.t. Western blot analysis showed a significant decrease in AC66 protein levels upon Ac-miR-5 exogenous overexpression (Fig. 3b, c), further confirming that *ac66* is a target of Ac-miR-5.

### Ac-miR-5 downregulates *ac66* at both mRNA and protein expression levels

**Fig. 4.**
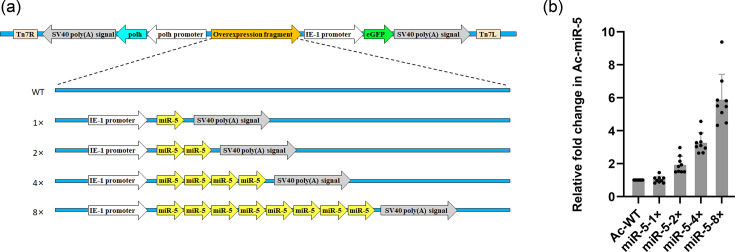
The construction of over-expression Ac-miR-5 viruses. (**a**) Schematic diagram of the reconstructions, 1×, 2×, 4× and 8× Ac-miR-5 viruses, as well as the control WT viruses. The yellow arrows represent the copies of the Ac-miR-5 precursor. Recombination was conducted using the Bac-to-Bac system. (**b**) RT-qPCR detection of the relative expression levels of Ac-miR-5 in recombinant viruses. The columns represent the fold change in Ac-miR-5 expression normalized to that of 5S rRNA expression. RNA samples were isolated from infected Sf9 cells at 9 h p.i.

To investigate how Ac-miR-5 regulates *ac66* expression in host cells, we generated four recombinant viruses expressing varying levels of Ac-miR-5. These constructs contained 1×, 2×, 4× and 8× additional copies of the precursor miR-5 sequence downstream of an *ie-1* promoter, alongside a WT recombinant virus control, using the Bac-to-Bac system (Fig. 4a). Ac-miR-5 expression levels in Sf9 cells infected with these viruses (WT, 1×, 2×, 4× and 8×) were assessed by stem-loop RT-qPCR (Fig. 4b). These results confirmed Ac-miR-5 expression from the recombinant overexpression viruses, enabling analysis of the biological effects of elevated endogenous Ac-miR-5 levels. Although the highest Ac-miR-5 expression was observed in the 8× virus (Fig. 4b), the 4× virus was selected for subsequent functional studies based on stability and efficacy assessments.

**Fig. 5.**
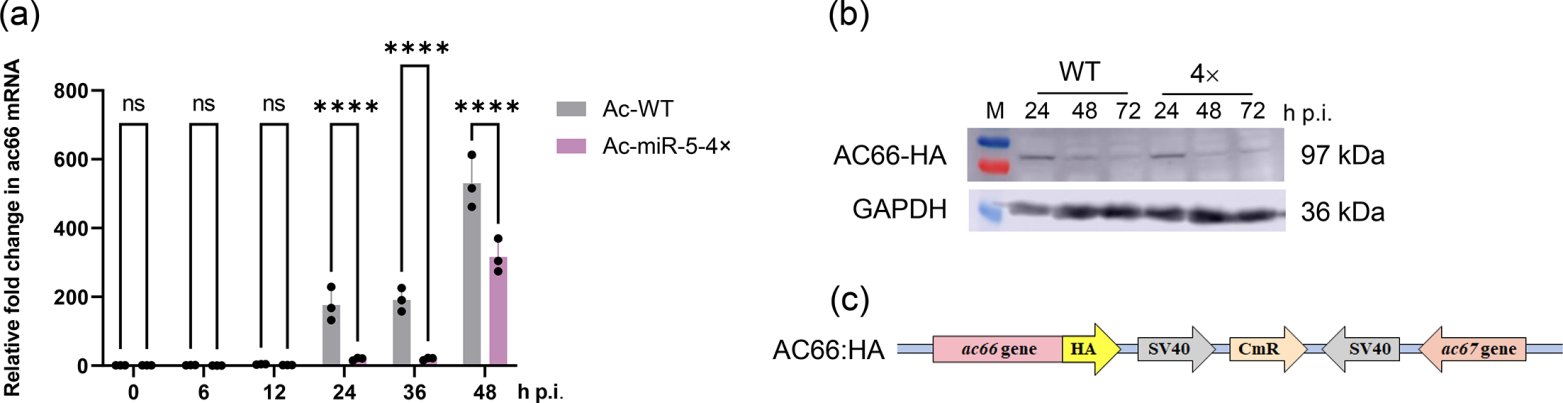
Analysis of the regulatory roles of Ac-miR-5 on *ac66*. (**a**) RT-qPCR detection of the expression levels of *ac66* mRNA. RNA samples were isolated from Sf9 cells at the indicated time points (10 p.f.u./cell). The columns represent the fold change in *ac66* mRNA levels. 5S rRNA was used as a reference gene. Each reaction was performed in triplicate for three independent experiments. (**b**) Western blot detection of the protein expression levels of AC66 under WT or 4× miR-5 recombinant virus infection. (**c**) Diagram of adding HA tag to Ac-WT and miR-5-4× recombinant viruses.

We first analysed *ac66* mRNA expression in infected Sf9 cells by RT-qPCR. *ac66* mRNA levels were significantly reduced at 24, 36 and 48 h p.i. in cells infected with the 4× virus compared to the WT virus (Fig. 5a), indicating that Ac-miR-5 can downregulate *ac66* mRNA expression.

Next, we examined the impact of Ac-miR-5 overexpression on AC66 protein levels. Recombinant viruses expressing HA-tagged AC66 (WT-66:HA and 4×-66:HA) were constructed based on the Ac-miR-5 overexpression constructs (Fig. 5c). Western blot analysis revealed the strongest AC66 bands at 24 h p.i., with a gradual decline thereafter in both WT and 4× virus infections. While AC66 protein levels of 4× virus appeared slightly lower at 48 h p.i. and slightly higher at 72 h p.i., no distinct difference was observed between the two treatments at 24 h p.i. (Fig. 5b).

Collectively, these results suggest that Ac-miR-5 downregulates *ac66* at both the mRNA and protein levels, although the changes in protein levels are less consistent across time points.

### Overexpressing Ac-miR-5 reduces BV production

**Fig. 6.**
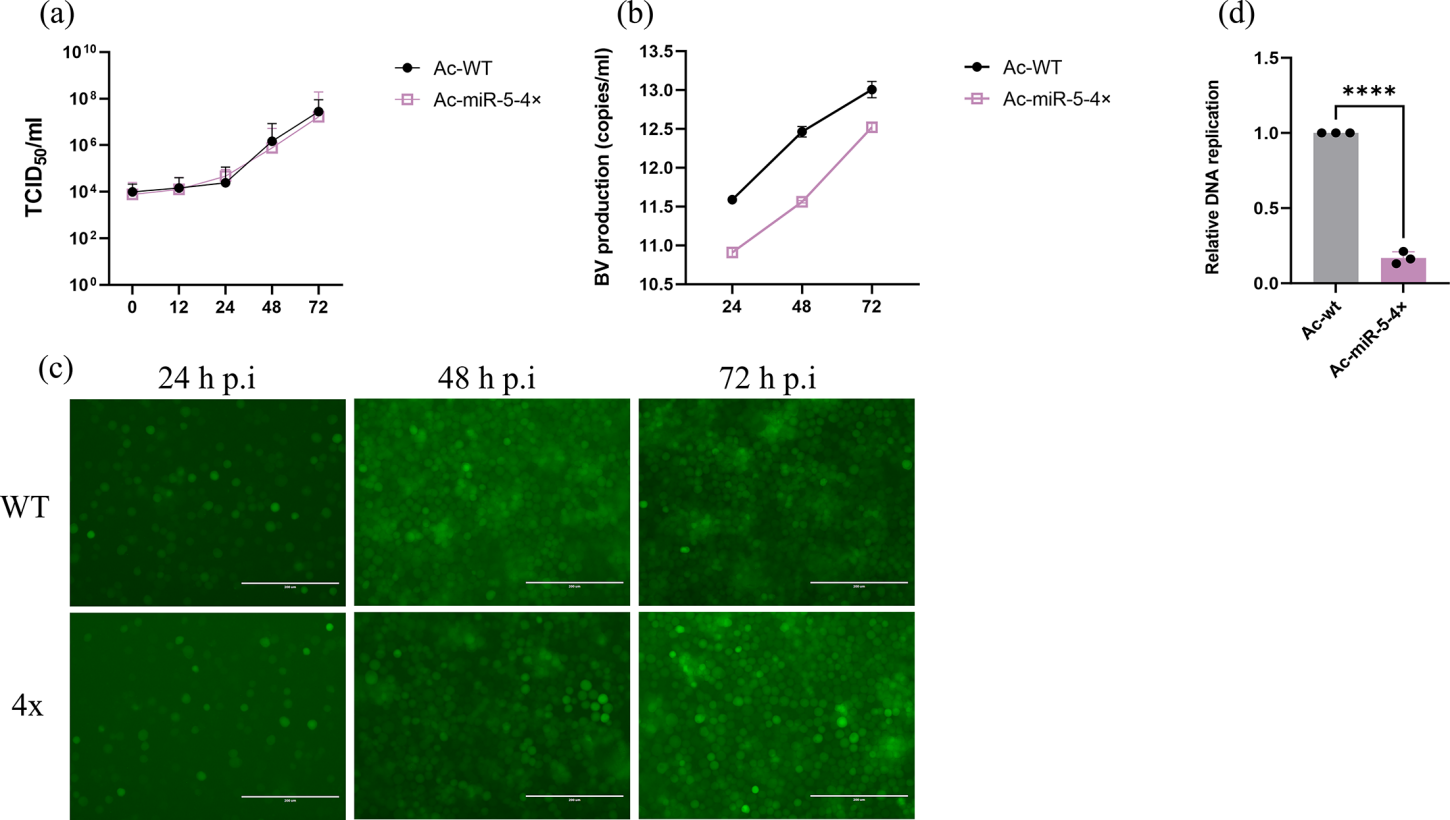
The effects of overexpressing Ac-miR-5 on BV production and viral DNA replication. Virus growth curve generated from WT or 4× virus-infected Sf9 cells. (**a**) Cell culture supernatants were harvested at the indicated time points, and BV titres were determined by TCID_50_ endpoint dilution assay. (**b**) BV production was determined by qPCR at 24, 48 and 72 h p.i. (**c**) Fluorescence microscopy observation of WT or 4× virus-infected Sf9 cells at 24, 48 and 72 h p.i. (**d**) Relative amounts of DNA replication were quantified by qPCR. DNA samples were generated from Sf9 cells infected with WT or 4× virus at 24 h p.i. Each reaction was performed in triplicate for three independent experiments. The data are presented as means and sd. ns, no significant; *P<0.05; **P<0.01; ****P<0.0001.

To investigate the influence of Ac-miR-5 on BV infectivity and production, Sf9 cells were infected with Ac-WT and Ac-miR-5-4× viruses at an MOI of 10 p.f.u./cell. TCID_50_ endpoint dilution assays revealed no significant difference in infectious BV titres between the Ac-miR-5 overexpression and WT control groups over a time course of 12, 24, 48 and 72 h p.i. (Fig. 6a). However, qPCR analysis of total BV production showed a slight decrease in the Ac-miR-5-4× virus group compared to the Ac-WT group at 24, 48 and 72 h p.i., by 5.86%, 7.24% and 3.74%, respectively (Fig. 6b). Consistent with the TCID_50_ results, monitoring of GFP fluorescence revealed no distinct difference in fluorescence spread between cells infected with the 4× virus and those infected with the WT virus from 24 to 72 h p.i. (Fig. 6c), further suggesting that Ac-miR-5 overexpression does not significantly affect infectious BV production.

These results differ from those previously reported for *ac66* knockout studies [23,24], likely because Ac-miR-5 does not perfectly match the *ac66* target site sequence (Fig. 2c). Consequently, its regulatory effect on *ac66* may be less pronounced, resulting in a partial inhibition of *ac66* expression rather than the complete loss of function observed in *ac66* knockout experiments.

Collectively, these findings indicate that Ac-miR-5 overexpression results in a mild reduction in total BV production without significantly affecting infectious BV production.

### Overexpressing Ac-miR-5 negatively affects viral DNA replication

To assess the impact of Ac-miR-5 on viral DNA replication, Sf9 cells were infected with WT and miR-5-4× viruses at an MOI of 10 p.f.u./cell. Total cellular DNA isolated at 24 h p.i. showed reduced viral DNA replication in miR-5-4×-infected cells compared to WT-infected cells by 83% (Fig. 6d), suggesting a negative impact of Ac-miR-5 overexpression on viral DNA replication. This decrease in DNA replication could be a consequence of the observed reduction in total BV production – as fewer BV could lead to reduced cell infection and thus lower viral DNA replication. However, the lack of a significant reduction in infectious BV and a slight reduction in total BV production suggest that the observed decrease in DNA replication may also be due to Ac-miR-5 overexpression affecting other as-yet-unknown factors directly involved in viral replication.

### Overexpressing Ac-miR-5 negatively affects the production of polyhedra

To further investigate the effect of Ac-miR-5 on viral infection, we examined polyhedra formation and ODV embedding in Sf9 cells using microscopy.

Light microscopy revealed a decrease in the number of cells containing polyhedra in the Ac-miR-5 overexpression group compared to the WT group. This decrease was particularly evident at 24 and 48 h p.i., with reductions of 51% and 27%, respectively. An 18% reduction was also observed at 72 h p.i., although this difference was not statistically significant by U test analysis (Table 1). The observed trend suggests that the difference in the number of polyhedra-containing cells between the two groups gradually diminishes as the infection progresses, even though the total number of polyhedra-containing cells increases in both groups. This may indicate that the effect of Ac-miR-5 overexpression on polyhedra formation gradually weakens over time.

**Fig. 7.**
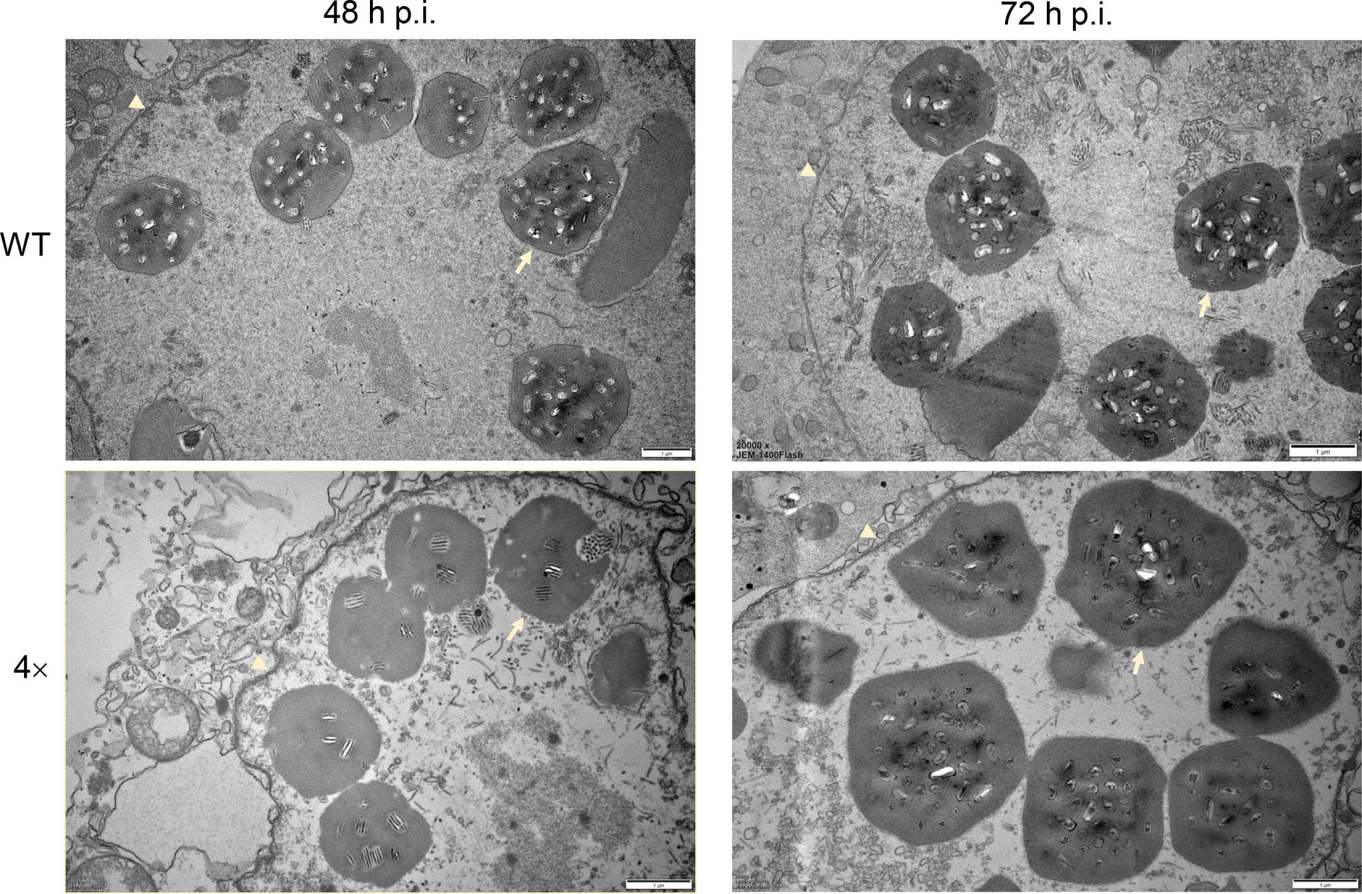
Electron microscope observation of the effects of overexpressing Ac-miR-5 on Sf9 cells. The cells were infected with WT and miR-5-4× viruses at 48 and 72 h p.i., respectively. The pale yellow arrows represent polyhedra, and the pale yellow triangles represent nuclear membranes.

Transmission electron microscopy revealed no significant differences in overall viral morphogenesis between cells infected with the Ac-miR-5 overexpression virus and the WT virus (Fig. 7). However, at 48 h p.i., polyhedral maturation appeared slightly delayed in the 4× virus group compared to the WT control. This delay was indicated by a slightly lower number of nuclear polyhedra and reduced embedded ODV. By 72 h p.i., the morphology and number of polyhedra were largely indistinguishable between the two groups.

Previous *ac66* knockout studies [23,24] have demonstrated that AC66 is essential for nucleocapsid transport from the nucleus to the cytoplasm. Disruption of this transport prevents efficient BV budding and formation [23,25]. While Ac-miR-5 overexpression in our experiments did not result in the complete loss of AC66 function observed in *ac66* knockout studies, the partial inhibition of *ac66* expression may nonetheless affect nucleocapsid trafficking, potentially contributing to the observed reductions in both BV and polyhedra production.

Baculoviruses produce two distinct types of progeny virions: BV and ODV. BV is essential for systemic transmission, while ODV is required for inter-insect transmission [6]. Nucleocapsids for both virion types are assembled within the infected cell nucleus, subsequently egressing to the cytoplasm where BV particles bud from the plasma membrane. Alternatively, nucleocapsids can be retained within the nucleus to form ODV, which are then embedded within polyhedrin protein crystals to form polyhedra [6,25]. Integrating these established processes with our experimental findings, we infer that Ac-miR-5 overexpression contributes to viral load reduction during the early and middle stages of infection. The observed rebound in polyhedra production during later stages suggests that this initial reduction in viral load may serve to mitigate host immune responses, ultimately facilitating successful viral establishment and propagation within the host.

### Overexpressing Ac-miR-5 negatively affects the mRNA expression levels of very late genes *polh* and *p10*

**Fig. 8.**
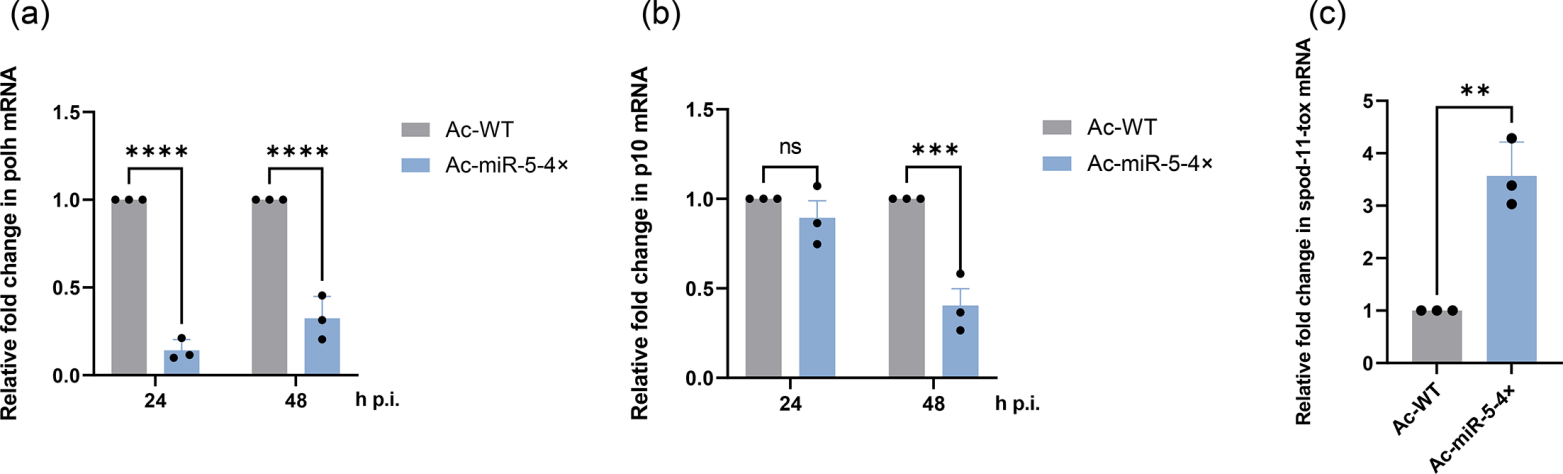
The effects of overexpressing Ac-miR-5 on the mRNA expression levels of viral very late genes and host immune-related genes. The mRNA expression levels of *polh* (**a**), *p10* (**b**) and *spod-11-tox* (**c**) were detected by RT-qPCR. The columns represent the fold change in these genes’ mRNA levels. The RNA levels were normalized to that of WT. 5S rRNA was used as a reference gene. The RNA samples were isolated from WT or 4× virus-infected Sf9 cells, for *polh* and *p10* at the indicated time points, for *spod-11-tox* at 6 h p.i. Each reaction was performed in triplicate for three independent experiments. The data are presented as means and sd. ns, no significant; **P<0.01; ***P<0.001; ****P<0.0001.

We also analysed the mRNA expression levels of the viral very late genes *polh* and *p10*, which are involved in polyhedra assembly [6,26, 27]. RT-qPCR analysis revealed that *polh* mRNA levels were significantly decreased at both 24 and 48 h p.i. in Sf9 cells infected with the Ac-miR-5 overexpression virus compared to the WT virus by 86% and 68%, respectively (Fig. 8a). *p10* mRNA expression was significantly reduced only at 48 h p.i. by 60% in the Ac-miR-5 overexpression group (Fig. 8b). This reduction in *polh* and *p10* mRNA levels may be a consequence of the observed decrease in viral DNA replication. Furthermore, the reduced expression of these two polyhedra-related genes likely contributes, at least in part, to the observed decrease in the number of polyhedra formed.

### Overexpressing Ac-miR-5 and host immune response

We also investigated the mRNA expression levels of *spod-11-tox*, a host immune-related gene [28,29]. RT-qPCR results showed a significant increase in *spod-11-tox* mRNA levels in Sf9 cells infected with the miR-5-4× virus at 6 h p.i. compared to the control (Fig. 8c). This early increase may represent the host cell’s defence response to viral infection. While *spod-11-tox* is likely not directly regulated by miR-5 at 6 h p.i., given that its target gene *ac66* is a late gene, an upregulation of host immune-related genes suggests that host cells are more responsive to viral infection. This heightened response could be an indirect consequence of miR-5 overexpression influencing other unknown viral or cellular factors.

## Concluding remarks

Ac-miR-5, a conserved alphabaculovirus miRNA, downregulates the viral gene *ac66* at both the mRNA and protein levels.

Considering that AC66 is associated with both BV and ODV [30] and is essential for nucleocapsid transport from the nucleus to the cytoplasm [23,24]. The observed effects of Ac-miR-5 overexpression on viral infection – namely, reduced BV production, DNA replication and polyhedra formation – are likely mediated, at least in part, by *ac66* downregulation. Specifically, Ac-miR-5-mediated suppression of *ac66* likely impacts BV production, which consequently limits subsequent cell infection. This reduction in cell infection, in turn, may restrict viral DNA replication and ultimately affect polyhedra formation. Thus, like the four previously characterized AcMNPV-encoded miRNAs [14,17], Ac-miR-5 contributes to viral load control, potentially mitigating host defences in the early to middle stages of infection to promote viral establishment and propagation. It is not uncommon for virus-encoded miRNAs to participate in the regulation of host immunity by targeting both viral and host genes to facilitate the propagation of the virus itself [31,32]. Furthermore, similar to miR-2 and miR-4, miR-5 appears to delay polyhedral formation, in contrast to miR-1 and miR-3, which promote it. The varying effects of AcMNPV-encoded miRNAs on polyhedra may be linked to the functions of their regulated target genes and whether those genes are upregulated or downregulated.

Finally, Ac-miR-5 may also target other as-yet-undiscovered genes, including cellular genes. Its overall impact on viral infection likely represents a complex interplay of regulatory effects on multiple targets.

## Supplementary material

10.1099/jgv.0.002155 Uncited Supplementary Material 1.

## Abbreviations

Ac-miR-5: AcMNPV-miR-5
AcMNPV: Autographa californica multiple nucleopolyhedrovirus
BmNPV: Bombyx mori nucleopolyhedrovirus
BV: budded virus
CT: threshold cycle
HA: haemagglutinin
h p.i.: hour post-infection
h p.t.: hour post-transfection
MFE: minimum free energy
miRNAs: microRNAs
NC: negative control
ODV: occlusion-derived virus
qPCR: quantitative real-time PCR
RT-qPCR: reverse transcription quantitative PCR
Sf9: Sf21-AE clonal isolate 9
