# Supplementary material for: An Autographa californica multiple nucleopolyhedrovirus-encoded microRNA, AcMNPV-miR-5, downregulates the expression of viral gene ac66
Source: J Gen Virol. 2025 Sep 30;106(9):002155. doi: 10.1099/jgv.0.002155 (PMC12483761; doi:10.1099/jgv.0.002155)
Supplement: Uncited Supplementary Material 1. [file jgv-106-02155-s001.pdf]

Table S1 Primers and oligonucleotides used in this paper (5’-3’)

Real time PCR primers	
stem-loop RT primer	GTCGTATCCAGTGCAGGGTCCGAGGTATTCGCACTGGATACGACTTCATG
Ac-miR-5	F: GCGTAGACGATGCCGTGCT<br>R: AGTGCAGGGTCCGAGGTATT
5S rRNA	F: GCCAACGTCCATACCACGTTG<br>R: GTCACCCATCCAAGTACTGACT
gp41	F: CGTAGTGGTAGTAATCGCCGC<br>R: AGTCGAGTCGCGTCGCTTT
actin	F: TGGGTATGGAATCTTGCG<br>R: GGAGCGATGATCTTGGTCT
ac66	F: TTCGTTGAAGTGTTAATCAGGATCG<br>R: GTTCGCATCACCAAATAAAAGACTC
p10	F: TTTTAGACGCCGTTAC<br>R: GCTTAGGCTTTAGTGAG
polh	F: CCCCGACTATGTACCTCA<br>R: ATTTCCTCCTCTTCAGCA
spod-11-tox	F: GCTGACAGTTGTGACCTCAGG<br>R: GGAGCGGAGTTTGCGTCAAC
Primers used for verification of target genes	
psicheck2-ac9	F: ACGCTCGAGATTTGCCGTCTGAAATGTTACC<br>R: TATGCGGCCGCTTGTTTTGTAATAAAGGTTCGACGT
psicheck2-ac15	F: ACGCTCGAGAATTGCTTTTGCTAAACCTGCA<br>R: TATGCGGCCGCGATTGTAGTCCGCCTTGC
psicheck2-ac21	F: TATGCGGCCGCCTAGAACTCGTTGTGGTTGCC<br>R: TATGCGGCCGCCTAGAACTCGTTGTGGTTGCC
psicheck2-ac65	F: ACGCTCGAGTTTCAATTGAACACAATCAGTAGTTACTATT<br>R: TATGCGGCCGCTGAGATTAGAAATACCCGCTGC
psicheck2-ac66	F: ACGCTCGAGCGAACGTCGCGGG<br>R: TATGCGGCCGCTGGGTCAATTGGTCAAAAGTG
psicheck2-ac109	F: ACGCTCGAGAGGGCGACGTGCCAT<br>R: TATGCGGCCGCCTAAAACCTCCGTCCGTTAAGA
psicheck2-ac125	F: ACGCTCGAGCAGACTTTTGCGCGACAACAAC<br>R: TATGCGGCCGCATTATCTTTACAACAATTAAAGCAATTAAAACAATTT
psicheck2-ac127	F: ACGCTCGAGCACAATTTAAATGAAATTATTAATAAAAACCAAAACGAT<br>R: TATGCGGCCGCTGATGGCTTCGAACGCTG
psicheck2-ac131	F: ACGCTCGAGGCTCGTTCGCCGAC<br>R: TATGCGGCCGCAAAATAACATCTTTAGCGGTTTCCAA
psicheck2-ac132	F: ACGCTCGAGTAGAAAATGAACAAAAACGATTGTATCC<br>R: TATGCGGCCGCAATTGCATTTCATCAAACAATTTTTTCATATAC
psicheck2-ac138	F: ACTGCGATCGCATGGCGGTTTTAACAGCCGTCGATTTAAC<br>R: TATGCGGCCGCTGGACACGACGCCGGTTTCGTGAAACGG

pCMV-ac66	F: ATTTGCGGCCGCGCAGCGATGGCCCAAATATGG<br>R: TGCTCTAGACTATTCGACGTTTGGTTGAACGCTG
miRNA and mimic sequences	
miR-5	UAGACGAUGCCGUGCUCAUGAA
miR-5 mimic	UAGACGAUGCCGUGCUCAUGAA
NC mimic	UUUGUACUACACAAAAGUACUG
